# *In-vivo* Activity of IFN-λ and IFN-α Against Bovine-Viral-Diarrhea Virus in a Mouse Model

**DOI:** 10.3389/fvets.2020.00045

**Published:** 2020-02-05

**Authors:** María Eugenia Quintana, Lucas José Barone, Myrian Vanesa Trotta, Cecilia Turco, Florencia Celeste Mansilla, Alejandra Victoria Capozzo, Nancy Patricia Cardoso

**Affiliations:** ^1^Centro de Investigaciones en Ciencias Veterinarias y Agronómicas, Instituto de Virología e Innovaciones Tecnológicas, Instituto Nacional de Tecnología Agropecuaria, Buenos Aires, Argentina; ^2^Consejo Nacional de Investigaciones Científicas y Técnicas, Buenos Aires, Argentina

**Keywords:** bovine-viral-diarrhea virus, mouse model, interferon-λ, interferon-α, antiviral activity

## Abstract

Bovine-viral-diarrhea virus (BVDV) can cause significant economic losses in livestock. The disease is controlled with vaccination and bovines are susceptible until vaccine immunity develops and may remain vulnerable if a persistently infected animal is left on the farm; therefore, an antiviral agent that reduces virus infectivity can be a useful tool in control programs. Although many compounds with promising *in-vitro* efficacy have been identified, the lack of laboratory-animal models limited their potential for further clinical development. Recently, we described the activity of type I and III interferons, IFN-α and IFN-λ respectively, against several BVDV strains *in-vitro*. In this study, we analyzed the *in-vivo* efficacy of both IFNs using a BALB/c-mouse model. Mice infected with two type-2 BVDV field strains developed a viremia with different kinetics, depending on the infecting strain's virulence, that persisted for 56 days post-infection (dpi). Mice infected with the low-virulence strain elicited high systemic TNF-α levels at 2 dpi. IFNs were first applied subcutaneously 1 day before or after infection. The two IFNs reduced viremia with different kinetics, depending on whether either one was applied before or after infection. In a second experiment, we increased the number of applications of both IFNs. All the treatments reduced viremia compared to untreated mice. The application of IFN-λ pre- and post-infection reduced viremia over time. This study is the first proof of the concept of the antiviral potency of IFN-λ against BVDV *in-vivo*, thus encouraging further trails for a potential use of this cytokine in cattle.

## Introduction

Bovine-viral-diarrhea virus (BVDV) is a single-stranded RNA virus that belongs to the family Flaviviridae, genus *Pestivirus*—along with border-disease virus and classical swine-fever virus—known to infect a wide range of wild and domesticated ruminants and porcine species (1, 2). BVDV is divided into two principal species, both including viruses of two biotypes, cytopathic, and noncytopathic (ncp), according to their activity in cultured cells. Acute ncp-BVDV infection is associated with diarrhea, respiratory disease, hemorrhagic syndrome, abortion, the weak-calf syndrome, teratogenic effects on fetuses, and mucosal disease (3, 4). The virus also causes severe immunosuppression that predisposes the infected animal to opportunistic pathogens. Moreover, persistence can be developed by *in-utero* infection with a ncp strain. Persistently infected (PI) animals are immunotolerant to the infecting strain and continuously disperse the virus within the herd.

BVDV is transmitted with high efficiency within infected herds, resulting in outbreaks and clinical disease that negatively affect production parameters. Vaccination applied to persistent-infection-free herds constitutes the only efficient tool for controlling BVDV. Nevertheless, even if vaccination is correctly applied and high-quality vaccines are used, development of adaptive immunity leaves a vulnerability window whose extent has not yet been defined. Vaccine failure is also favored by the presence of PI animals, the lower efficacy of vaccines in animals with maternal immunity, and the emergence of new viral strains not included in the vaccine, among other issues. In this scenario, the use of an effective antiviral agent is paramount.

The type-I and type-III interferons (IFNs) are virus-induced cytokines that potently restrict viral replication during the first days of infection before activation of the adaptive immune system occurs (5, 6). The type-I IFN family consists of several IFN-α subtypes, a single IFN-β and several minor members that all bind to and act via the IFN-α/ß–receptor complex, expressed on most nucleated cells (5, 6) with the possible exception of intestinal epithelium (7, 8). The members of the type-III IFN family (IFN-λ1, IFN-λ2, and IFN-λ3) bind to a different receptor complex (the IFN-λ receptor), which is highly expressed on epithelial cells (5, 9). Although type-I and -III IFNs use different receptor complexes, both cytokines activate similar signal pathways (9, 10) and possess comparable antiviral activities (11), though toxicity is usually lower for IFN-λ because of its cell-type–restricted target. These IFNs have been tested *in-vivo* (12–18), revealing high non-specific antiviral activities; and although action of these cytokines is exerted in different cell types, no reports have appeared in the literature on experiments that evaluate the combined use of IFN-I and -III for the prophylaxis and/or therapeutic treatment of viral infections *in-vivo*.

BVDV is used as a model virus for human hepatitis C antiviral studies (19, 20); therefore, many antiviral agents have been developed (21). Human as well as bovine IFNs have been tested against BVDV *in-vitro* (22), but the *in-vivo* efficacy has been difficult to demonstrate. Most of the efforts in using IFNs as antiviral cytokines for cattle have focused on treating PI animals (23)—and with arguable success—but controlling acute infections *in-vivo* has not been assessed thus far. Moreover, circulating BVDV strains are ncp, which complicates measuring infectivity *in vitro*.

Viral mechanisms that counteract IFN-α activity have been identified, but, acute BVDV infection with ncp strains induces high levels of systemic IFN-α and other IFNs in the infected cattle (24), meaning that these IFNs may be functional within an *in-vivo* situation. To the best of our knowledge, no evidence has been garnered for the use of IFN-α or other IFNs to prevent and/or treat acute BVDV infection *in-vivo*. We have recently demonstrated that several BVDV strains are susceptible to bovine IFN-α and IFN-λ *in vitro*. We also developed a method to easily measure infectivity of BVDV by visualizing infected cells using a monoclonal antibody against a non-structural protein (NS3), considered a marker of virus replication (25). Recently, a mouse model of BVDV infection was set-up and tested with several strains showing that mice developed viremia without clinically manifest disease (26, 27). In this study, we made use of all these tools, set up the BALB/c infection model for 2-well characterized type-2 ncp BVDV strains (28) and evaluated the efficacy of prophylaxis and treatment with IFN-α and IFN-λ in reducing BVDV infection.

## Materials and Methods

### Cells and Viruses

The Madin-Darby bovine-kidney (MDBK) cell line from the American Type Culture Collection (ATCC) was provided by the Institute of Virology (INTA) Argentina. The cells were cultured in Dulbecco's Modified Eagle's Minimal Essential Medium (DMEM, Gibco) with 10% (v/v) gamma globulin-free, mycoplasma-tested and virus-screened fetal bovine serum (FBS, HI FBS Qualified, Gibco). FBS was also controlled for the BVDV genome by RT-PCR following a standard protocol (see below). The absence of live virus in the FBS was further corroborated by serial passage in MDBK cells followed by immunofluorescent staining as previously described (29).

Two genotype-2 ncp BVDV strains were used: the reference BVDV NY-93 high-virulent strain (GenBank accession number AF502399) and the low-virulent field strain isolated in the Buenos Aires province, Argentina (BVDV 98-124, GenBank accession number MH074881. Both strains had been previously characterized in a colostrum-deprived-calf model (28). BVDV stocks were produced by infecting MDBK cells, following standard procedures (30). Titers were 8.26 × 10^6^ and 5.24 × 10^6^ 50% tissue-culture–infective-dose (TCID50)/ml for the 98-124 and NY-93 strains, respectively.

### Animals

Female BALB/c mice, 6–8 weeks' old weighing 18–20 g were purchased from the animal facility of the School of Veterinary Science, University of Buenos Aires. All the feeding and experimental procedures were carried out under specific pathogen-free conditions at the animal experimental center of our institute (INTA). The animals were housed in a temperature- and light-controlled environment and had free access to food and sterile water *ad libitum*. After acclimatization to the light-dark cycle for 1 week, the experiment was started. The procedures with the mice were performed following national animal-welfare regulations (Protocol N° 6/2017 CICUAE, INTA).

### Antibodies

Fluorescein isothiocyanate- (FITC-) conjugated polyclonal commercial antibody against BVDV (VMRD BVDV Direct FA Conjugate, WA, USA) was used to visualize the presence of virus by immunofluorescence. The monoclonal antibody anti-NS3 was kindly provided by Dr. Gerrit Keizer (Prionics).

### Mouse-BVDV–Infection Model

Two groups of eight mice each were inoculated intraperitoneally (IP) with 0.4 mL of DMEM (Gibco) without serum, containing 1.25 × 10^6^ TCID50/ml of the BVDV NY-93 or 98-124 strains. Mock-infected mice (*n* = 4) were administered 0.4 mL of DMEM. The virus stocks were produced according to the procedures described above but with culture media without FBS. The animals' weights and body temperatures were controlled during the experiment. Whole-blood and serum samples were taken at the beginning of the experiment and at 2, 4, and 7 days post-infection (dpi) and viremia assessed by In-Cell ELISA™. Proinflammatory cytokines were measured at 0, 2, and 4 dpi with a commercial kit (the BD® CBA inflammation kit). The mice were euthanized at 7 dpi and the heart, spleen, liver, kidney, mesenteric lymph nodes, and brains removed. Each organ was divided into two equal parts that were used for histopathological analysis and for virus isolation as described previously (28).

In a second experiment, two groups of five mice each were infected with the 98–124 strain, or mock-infected, and sampled at 0, 4, 7, 10, 14, 21, 35, 43, and 56 dpi. The sera were aliquoted and stored at −80°C until use. The mice were euthanized at the end of the experiment and the spleen, liver, and mesenteric lymph nodes removed and processed for histopathology and RT-nested PCR, as detailed below.

### Prophylaxis and Treatment With IFNs-Experimental Design

Experiment 1: Thirty-seven BALB/c mice were randomly divided into eight groups. Each group received recombinant mouse IFN-α (250,000 U/dose, Miltenyi Biotec^®^, Alemania) or IFN-λ (2 μg/dose, Sigma^®^) by subcutaneous injection. The latter were selected on the basis of previous reports (31–34). BVDV 98–124 was inoculated IP, as described above. The IFNs were administered the day before infection (−1 dpi: i.e., the “pre-infection” groups, *n* = 5 each) or the day after infection (+1 dpi, “post-infection” groups, *n* = 5 each). Mock-treated mice received PBS (the buffer used to dilute the IFNs) on −1 and also +1 dpi (*n* = 5). The mock-infected mice received DMEM at 0 dpi (*n* = 6), these animals were not treated with IFNs. Another two groups of three animals each that were inoculated with each IFN but not infected were used as the treatment controls.

Experiment 2: Forty BALB/c mice were randomly divided into eight groups of five animals each. IFNs were administered at the same doses as in Experiment 1. Each group received the IFNs before (−2 and −1 dpi) or after (+1 and +2 dpi) the infection. The mock-treated group received PBS both before and after infection. Infection was carried out at 0 dpi with the mock-infected mice receiving DMEM at the same time point (the naïve group). The serum samples were collected at 0, 2, 4, 7, 10, 15, 20, and 30 dpi and stored at −20°C until use.

### Viremia

Viremia was determined in individual- or pooled- serum samples added to MDBK cells monolayers by revealing virus infectivity with a monoclonal antibody against NS3 (25). The protocol of this so-called “In-Cell ELISA,” has been described previously and was applied with the following modifications. To MDBK cells grown in 96-well culture plates (1.5 × 10^3^ cells/well), 50 μl of serial 2-fold dilutions (1/4–1/320) of serum were added to duplicate wells before incubation at 37°C, for 1 h in an atmosphere of 5% CO_2_. The inoculum was then removed by washing the cells with PBS 1x and the plates incubated for 48 h at 37°C−5% CO_2_. The monolayers were fixed with 4% (v/v) formaldehyde in PBS for 20 min, permeabilized with 0.01% (v/v) Triton X-100 in PBS for 20 min at room temperature, and finally blocked with 10% (v/v) equine serum in PBS for 90 min. The resulting supernatants were then gently aspirated and the primary antibody (mAb anti-NS3, Prionics) was added followed by incubation for 1 h at 37°C. The reaction was revealed with an anti-mouse peroxidase conjugate and the substrate 2,2′-azino-bis (3-ethylbenzothiazoline-6-sulfonic acid) [ABTS]. To account for differences in cell seeding, the density was normalized according to the readings at 595 nm after staining with the dye Janus-Green (SIGMA, San Luis, MO; at 50 μl/well). Once normalized, the ABTS-OD_450_ values of each sample were subtracted from those of the mock-treated wells to give the corrected OD_450_ values, which were then averaged. For NS3 titration, the inverse of the last dilution giving a value over the cut-off of the assay (OD = 0.2) was informed.

### Cytokine Measurement

IFN-γ, MCP-1, IL-6, IL-10, and TNF-α, were quantified in serum samples using the commercial CBA inflammation kit (BD^®^) according to the manufacturer's instructions. Briefly: Samples were diluted 1:2 in assay diluent then mixed with 60 μl of the Capture Beads Mix from the kit. Fifty microliters of Mouse Inflammation PE Detection Reagent was added and incubated for 2 h in the dark, at room temperature. The cells were next washed twice, centrifuged at 200 *g* for 5 min, and finally analyzed by flow cytometry.

Alternatively, commercial TNF-α- and IL-10–precoated sandwich-ELISA kits were used (BD Pharmingen, San Diego, CA) according to the manufacturer's instructions. Briefly, 100 μl of capture Antibody were added per well and incubated overnight at 4°C. After three washes, the plates were blocked with 10% (v/v) FBS in PBS and incubated for 1 h at room temperature. The samples were diluted 1:2 in 10% (v/v) FBS in PBS and 100 μl added to each well followed by an incubation for 2 h at room temperature. After washing, the plates were incubated with the corresponding detection solution of antibody plus streptavidin-bound horseradish peroxidase, and TMB (BioSite TM 4125). The reaction was stopped with 2 N H_2_SO_4_ and plates read at 450 nm.

### RT Nested PCR

RNA was extracted from whole blood and minced organs using a commercial kit (High Pure RNA Tissue Kit, Roche) following the manufacturer's instructions. RNA samples were quantified in a NanoDrop^®^ (Thermo Fisher Scientific, Wilmington, DE). The values for the ratio of absorbance at 260/280 nm and 260/230 nm were considered an assessment of the purity of the extracted RNA. The reverse transcription was carried out with Moloney-murine-leukemia-virus reverse transcriptase and the rev-326 primer (35). The nested PCR was conducted in a conventional thermocycler (T-18, Ivema SRL, Argentina) following a protocol developed by Jones et al. (36). The first round involved the use of the Pan-Pestivirus 324 and 326 primers (35) at 300 nM, the second round the Pesti-3 and Pesti-4 primers (37) at the same concentration. The reaction products were analyzed by electrophoresis in a 1.5% (w/v) agarose gel and compared with a ladder of molecular-weight markers (Cien Marker, Biodynamics SRL). A band of 171 bp is expected for positive detection.

### Histopathology

The ablated organs (liver, spleen, heart, kidney, mesenteric lymph nodes, and brain) were immediately fixed in 10% (v/v) neutral buffered formaldehyde and embedded in paraffin. The fixed organs were sliced and stained with hematoxylin and eosin followed by histopathological analysis by microscopy.

### Data Analysis

Paired-comparisons were performed with the Mann-Whitney test. The results between different experimental groups with respect to the areas under the absorbance curves in **Figures 3C**,**D** and **Figure 5B** were compared by means of the Krustall-Wallis test, followed by the Dunn's multiple-comparisons test. In all instances, a confidence interval of 95% was considered. The analysis was performed with GraphPad Prism v5.0 (GraphPad Software, CA, USA).

## Results

### Setup of the BVDV-Mouse Model: Infection With Low- and High-Virulence Type-II Strains

In the present study, BALB/c mice were infected with 2-well characterized ncp BVDV–genotype-2 strains of different virulence (28), following recently published protocols (26, 27).

**Figure 1:**
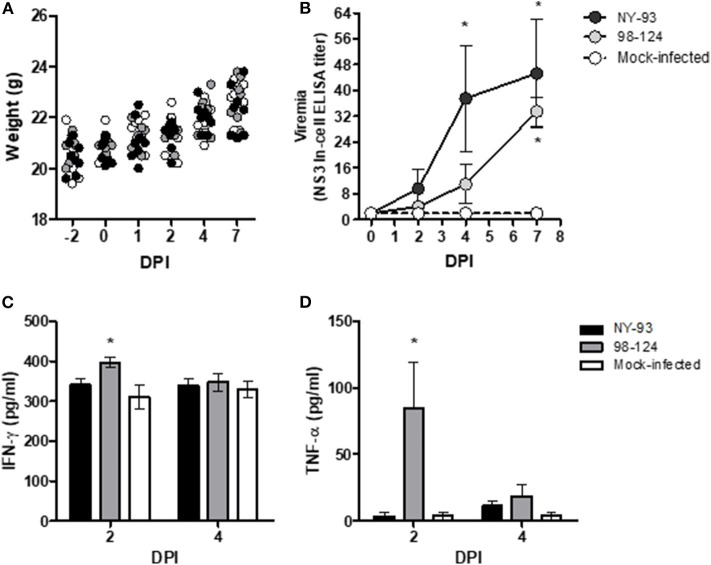
Mouse-BVDV–infection model. BALB/c mice were infected with two ncp BVDV genotype-2 strains, 98-124 and NY-93. In the figure, body weight **(A)**; viremia **(B)**; and the levels of the cytokines IFN-γ **(C)** and TNF-α **(D)** in pg/ml are plotted as functions of the time in days post-infection (DPI). Key to the point and bar textures: black, primary antibody against the NY-93 BVDV strain; gray, primary antibody against the 98-124 BVDV strain; white, mock-infected mice. The asterisks (*) denote values significantly higher than those measured in the mock-infected mice (*p* < 0.05).

In the first experiment, groups of eight BALB/c mice were inoculated IP with either BVDV strain or PBS. None of the mice exhibited any clinical signs of illness—neither changes in feeding patterns nor alterations in behavior associated with malaise. BVDV infection, furthermore, did not affect the mice's weight gain (Figure 1A) or modify their body temperature (data not shown) during the whole experiment. Viremia was assessed in serum samples by seeding each serum sample on MDBK cells and revealing virus replication with the NS3-In-Cell ELISA (Figure 1B). Viremia was first detected at 4 dpi for the high-virulence strain NY-93 and at 7 dpi for the field isolate 98-124. The IFN-γ levels increased significantly at 2 dpi (*p* < 0.05) in mice infected with strain 98-124 while in mice infected with NY-93 the increase that occurred was not significant (*p* = 0.052; Figure 1C). The low-virulence strain 98-124, induced an increase in the systemic levels of TNF-α at 2 dpi that decreased by 4 dpi (Figure 1D); and since the cytokine evidenced no change in the NY-93–bearing mice, the TNF-α levels were comparable in all three groups at that later time. The rest of the cytokines as well as the chemokine MCP-1 did not manifest significant differences between the groups (data not shown). Viral RNA was detected by RT-PCR in blood samples at 7 dpi in all infected animals. The results from the histopathological analysis did not detect any lesions that could be associated with the infection (data not shown).

**Figure 2:**
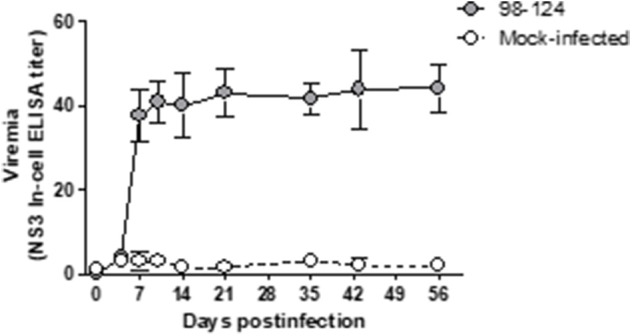
Kinetics of viral replication, as measured in the BVDV levels of blood samples of mice infected with the BVDV 98-124 strain by NS3-In-cell ELISA. Samples were taken at different time points from 0 to 56 days post-infection. Values are expressed as the mean NS3 titers ± SD of triplicates.

The infected mice did not resolve the viremia by a week's time post-infection; in fact, at that time high levels were achieved (Figure 1B). In order to complete the viremia curve, we monitored the infected animals for another 56 days. During this time the infected mice failed to evidence any clinical signs of the disease or underwent any change in weight or behavior compared to the naïve animals, although the virus remained in circulation for up to 56 dpi within levels similar to those measured at 7 dpi (Figure 2).

### Use of IFN-α and -λ for the Prophylaxis or Treatment of BVDV Infection

The activity of both IFNs was then assessed using BVDV 98-124 in the mouse model. This virus strain was selected because it is a regional representative of typical type 2 strain (28) and also due to the 2 dpi TNF-α systemic innate response induced by this strain that can be easily measured in sera.

**Figure 3:**
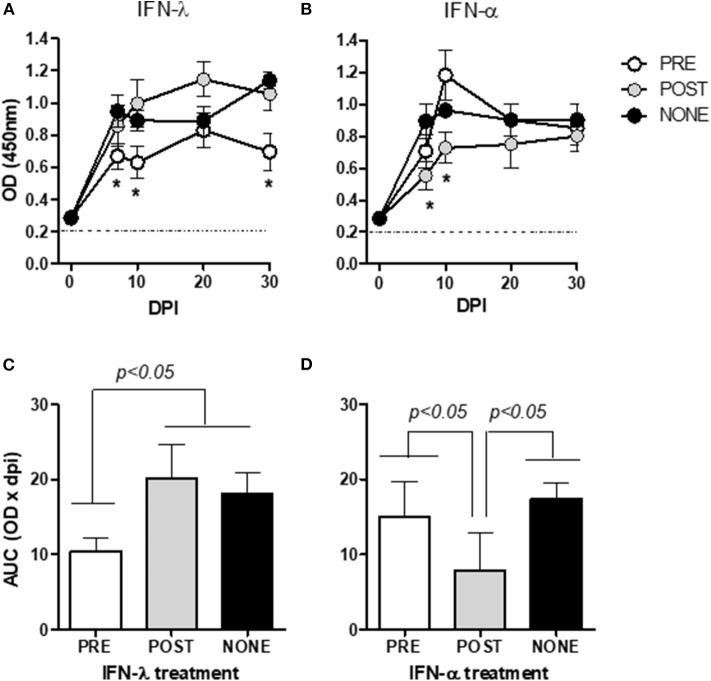
Effect of IFN prophylaxis or treatment in viremia. In the figures, the kinetics of viral replication, are expressed as mean OD_450_ ± SD of triplicate values. Mice were inoculated with either IFN-λ **(A)** or IFN-α **(B)** before (PRE, white circles) or after (POST, gray circles) infection, or infected but left untreated (NONE, black circles). The figures of **(C)** (IFN-λ) and **(D)** (IFN-α) are plots of the areas under the respective curves from **(A,B)** (mean ± SD) for the three inoculation protocols (PRE, POST, and NONE). The corresponding values for the noninfected mice (mock-infected groups) were below the detection levels of the assay and are thus not included in the figures. The asterisks (*) in **(A,B)** denote values significantly lower than those measured in the NONE group (*p* < 0.05). The dotted horizontal line in figures **(A,B)** depict the cut-off value. DPI, days post-infection.

Type-I and -III IFNs were applied either before or after infection (Figure 3). A peak of viremia was observed at 7 dpi in the infected, untreated mice (PBS group) as seen above (Figure 1B), whose levels thereafter were maintained for up to 30 dpi. Mock-infected animals, either treated or not with IFNs, did not develop viremia and no signs of discomfort or changes in behavior, temperature and weight gain were observed due to the IFN-treatment (data not shown). IFN-λ applied before infection (Figure 3A) reduced the viremia to values significantly lower than those observed in the PBS group at 7, 10, and 30 dpi (*p* = 0.0307, 0.0317, and 0.0378, respectively; *cf*. the asterisks in Figure 3A); whereas the application of IFN-λ post-infection had no effect in reducing virus levels with respect to that untreated group.

The treatment with IFN-α post-infection diminished the mean viremia values at 7 and 10 dpi (*p* = 0.0370 and 0.0159, respectively) compared to the PBS-treated mice (*cf*. the asterisks in Figure 3B). In contrast, the prophylactic application of IFN-α did not change the course of the viremia. By analyzing the area under the curves for each IFN we confirmed that a prophylaxis with IFN-λ (Figure 3C) or a treatment after infection with IFN-α (Figure 3D) significantly reduced the viremia compared to the infected, untreated mice.

### Combined Use of IFN-α and -λ

The combined use of both IFNs was then evaluated by duplicating the number of applications, pre- (at −2 and −1 dpi) and/or post-infection (at +1 and +2 dpi). Serum samples were collected at different times from 0 to 30 dpi.

**Figure 4:**
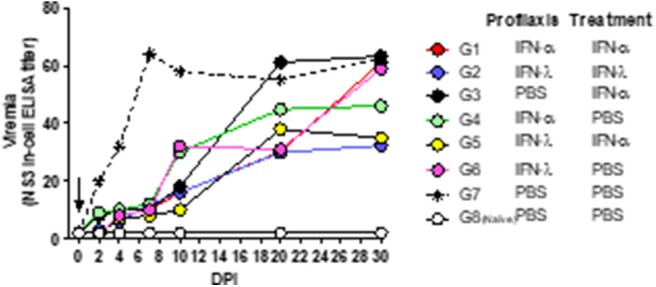
Effect of combined treatment in viremia. In the figure, the kinetics of viral replication in pools of serum, expressed as NS3 titers, is plotted as a function of the days post-infection (DPI). The arrow marks the time of infection. The key to the experimental groups to the right of the figure, summarizes the IFN-pre- and/or –post-infection protocol for each of the experimental groups (G1–G8).

Individual samples were pooled and tested by NS3-In-Cell ELISA (Figure 4). Infected, untreated mice (Group 7) developed a viremia that peaked at 7 dpi and continued within similar levels up to 30 dpi. All the IFN-treated mice evidenced a reduced viremia for up to 7 dpi with similar titers to those found in the naïve animals (Group 8). Four groups (1, 2, 3, and 5) maintained those low viral titers for up to 10 dpi, while the other two groups (4 and 6) manifested only a modest increase in virus titers. From 10 to 30 dpi, the viremia increased in almost all the groups to reach similar levels to those observed in infected-untreated animals (Group 7). At 30 dpi, only groups 2 and 5—and to some extent Group 4—exhibited titers lower than those of the untreated animals.

**Figure 5:**
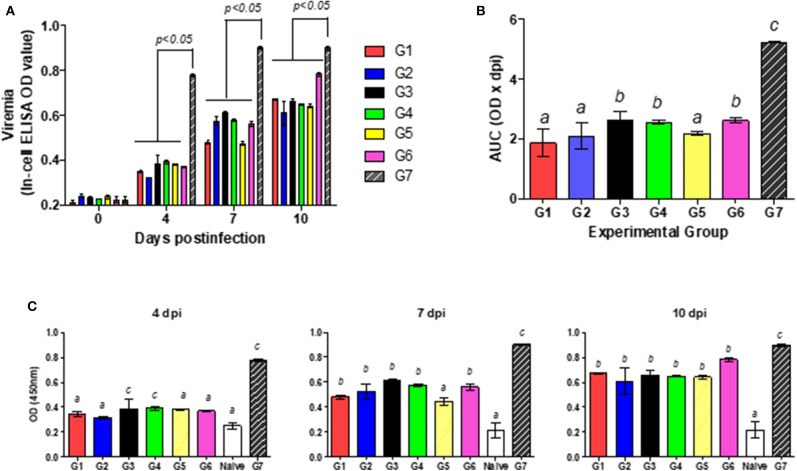
Effect of combined treatment in viremia. **(A)** Viral replication was measured as the mean OD_450_ levels ± the SD reflecting the NS3 titer, for each of the experimental groups G1–G7. **(B)** The mean value ± the SD for the area under the curve (AUC) resulting from the bar heights in **(A)** from days 1 through 10 is plotted for each of the experimental groups G1–G7. **(C)** Viral replication was measured as in **(A)** for each individual animal in the groups G1–G7 plus Group G8 (the naïve mice) at Day 4 (left figure), Day 7 (middle figure), and Day 10 (right figure) post-infection. In **(B,C)**, different letters above the bars mark significant differences between groups (*p* < 0.05).

Individual serum samples were then analyzed by the In-Cell-ELISA assay at 0, 4, 7, and 10 dpi. As described above, all the groups had NS3 levels significantly lower than the infected, untreated mice (*p* < 0.05; Figure 5A). Table 1 lists the maximum and minimum mean and the median values plus the standard deviations, standard errors, and coefficients of variation for the OD_450_ measured at 4 and 7 dpi. The coefficients of variation were low (ranging from 1 to 20%), indicating that all the animals within each group contained quite similar virus levels. In order to define which treatment was the most effective in reducing viremia, the area under each individual curve between 0 and 10 dpi was computed (Figure 5B). All the IFN-treated mice evidenced significant differences in mean area under the curve (AUC) values from that of the infected, untreated mice (*p* < 0.05), though groups 1, 2, and 5 were associated with the lowest AUC values.

**Table 1:** Statistical analysis of viremia values measures in the NS3-In-Cell ELISA assay at 4 and 7 dpi.

		**Groups/treatments**							
		**G1 IFN-α/IFN-α**	**G2 IFN-λ/IFN-λ**	**G3 -/IFN-α**	**G4 IFN-α/-**	**G5 IFN-λ/IFN-α**	**G6 IFN-λ/-**	**G7 -/-**	**G8 NAÏVE**
4 dpi	*n*	5	5	5	5	5	5	5	5
	Minimum	0.3420	0.3000	0.3410	0.3670	0.3710	0.3650	0.7670	0.2250
	Maximum	0.3670	0.3240	0.5010	0.4150	0.3870	0.3750	0.7920	0.2500
	Median	0.3485	0.3165	0.3500	0.3910	0.3820	0.3710	0.7780	0.2480
	Media	0.3515	0.3143	0.3855	0.3914	0.3802	0.3708	0.7790	0.2528
	SD	0.0116	0.0114	0.0771	0.0175	0.0069	0.0040	0.0089	0.0271
	SE	0.0058	0.0057	0.0385	0.0078	0.0031	0.0018	0.0040	0.0135
	CV	3.32%	3.64%	20.01%	4.47%	1.84%	1.09%	1.15%	13.44%
7 dpi	*n*	5	5	5	5	5	5	5	5
	Minimum	0.4620	0.4600	0.6050	0.5640	0.4120	0.5390	0.8870	0.2250
	Maximum	0.4950	0.5930	0.6230	0.5860	0.4870	0.5980	0.9130	0.2500
	Median	0.4780	0.5255	0.6120	0.5780	0.4395	0.5545	0.9010	0.2375
	Media	0.4806	0.5260	0.6130	0.5776	0.4445	0.5615	0.9002	0.2375
	SD	0.0133	0.0597	0.0085	0.0087	0.0325	0.0259	0.0098	0.0123
	SE	0.0059	0.0298	0.0042	0.0039	0.0163	0.0129	0.0044	0.0062
	CV	2.77%	11.36%	1.38%	1.50%	7.32%	4.62%	1.10%	5.20%

*SD, standard deviation; SE, standard error; CV, coefficient of variation*.

Viremia levels for all the IFN-treatments were compared with the naïve group by means of the one-way ANOVA. All the pre- and/or post-infection protocols tested resulted in significantly lower OD_450_ values than those of the infected, untreated mice of Group 7 at all three times dpi. Moreover, the values obtained with the animals from Group 2, treated with IFN-λ at both pre- and post-infection, did not yield significant differences from those of the naïve mice at 4 dpi (*p* > 0.05, Figure 5C); whereas on this same day the other IFN-schedules yielded higher NS3-values than those of the naïve animals, with no differences being found upon the use of those different protocols. None of the values for the treated groups, however, were comparable to those of the naïve animals at 7 and 10 dpi. The mice from group 5 exhibited lower NS3-OD_450_ values than did all the other INF-treated animals at 7 dpi whereas by 10 dpi the titers of all the groups were similar.

**Figure 6:**
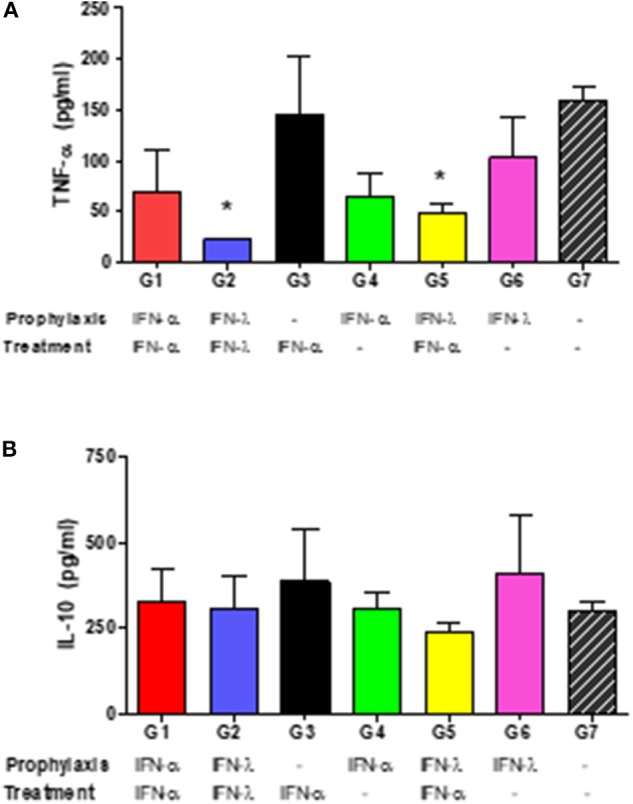
Systemic cytokine levels. In the figures, the mean concentrations ± SD of IFN-α **(A)** or IL-10 **(B)** in pg/ml of individual serum samples at 2 dpi are plotted for each experimental group. The asterisks (*) denote significant differences compared to the corresponding levels measured in the infected, untreated animals (Group 7) at a *p* < 0.05.

Systemic TNF-α and IL-10 levels were quantified in serum samples at 2 dpi. Infected, untreated mice (Group 7) responded to BVDV infection by secreting high levels of TNF-α (Figure 6A). Similar levels of this cytokine were measured in mice that received only one dose of IFNs, either IFN-λ as prophylaxis (Group 6) or IFN-α post-infection (Group 3). TNF-α levels in groups 1 and 4 (both received only IFN-α) were lower than those observed in Group 7, but the differences were not statistically significant (*p* = 0.14 and *p* = 0.09 for groups 1 and 4, respectively). A significant reduction in the systemic TNF-α levels (*p* < 0.05) was found in animals that received either IFN-λ pre- and post-infection (Group 2), or IFN-λ pre- and IFN-α post-infection (Group 5). By contrast, systemic IL-10 levels did not differ statistically between all the experimental groups (Figure 6B) although within the seven groups Group 5 displayed the lowest level for that cytokine.

## Discussion

Antiviral agents can be valuable tools to control BVDV in the field. An analysis of the activity of those agents against this virus is critical not only for the cattle industry but also for human health, as BVDV has been extensively studied as a model for Hepatitis-C virus (HCV). Because type-I and -III IFNs are produced in response to BVDV infection (38, 39), attempts have been made to treat PI animals with human IFN-α (23, 40); but no information has been reported on the efficacy of that cytokine in controlling acute BVDV infections *in-vivo*. Our study provides the first evidence of the anti-BVDV activity of both IFN-α and IFN-λ *in-vivo*. The present work is also the first time a murine model has been used to study the efficacy of an antiviral compound against BVDV.

The BVDV–BALB/c-mouse model was developed on the basis of procedures described by Seong et al., involving the IP-inoculation route, which resulted in more consistent viremia levels than intranasal inoculation (26). Our results indicate that mice can be infected by two type-2 BVDV field ncp strains of different virulence. The difference in virulence between the two strains was evidenced by an earlier onset of viremia in the mice infected with the NY-93 strain than in those bearing the 98-124 strain, and by the induction of a peak of systemic TNF-α in all the animals infected with 98-124. None of the infected mice developed clinical disease, coincident with previous reports (26, 41); with the infection being verified by only the presence of viremia, both by virus titration and RT-PCR.

We provided here two key contributions to the BVDV-BALB/c model-namely, (1) the use of the NS3-In-Cell ELISA to measure viremia by revealing virus infectivity directly on the MDBK infected cells using an antibody against NS3 and (2) the long-term follow-up of viremia. The NS3-In-Cell ELISA recently developed by our laboratory (25) can measure viral titers with a minimum amount (15 μl) of serum sample, enabling the daily bleeding of animals in accordance with standard animal-welfare regulations. Moreover, NS3 is a conserved non-structural protein used as a marker of viral replication for any BVDV strain, regardless their genotype or biotype. Following up viremia for almost 2 months after infection allowed us to confirm that the mice became chronically infected with BVDV. Another *flavivirus*, the Norway-rat hepacivirus (genus *Hepacivirus*), persisted in BALB/c mice for 6 weeks post-infection (42), whereas BVDV lasted for at least 8 weeks. Moreover, that the virus could be isolated only from the blood was interesting. Unpublished data from our laboratory analyzing the infection of murine splenocytes and cultured bone marrow-derived dendritic cells (DCs) have suggested that the DCs may be responsible for sustaining the infection in these animals. In fact, bovine DCs are not killed by BVDV infection (29). A role of the DCs in BVDV infection in mice—or even simply the extent of the action of those cells in persistent infection and the lack of IFN synthesis—needs to be confirmed by experiments aimed at this specific purpose. The role of both Erns, which degrades double-stranded RNA, and the capacity of N(pro) in promoting the degradation of the murine transcription factor IRF-3 which shares 68% of homology with the Bos Taurus orthologous, should be also explored as they have been associated with viral-induced immunotolerance in cattle (43).

We have recently reported the *in-vitro* antiviral activity of recombinant IFN-α and -λ with several BVDV strains of different biotype and genotype, including field strains (25). Human IFN-α has also prove to be effective against cp and ncp BVDVs strains (44). Mouse IFN-α is able to reduce more than 60% BVDV infection of mice splenocytes (unpublished data from our lab). Recombinant mouse IFNs have exhibited potent antiviral activity in infection models with numerous viruses such as herpes-simplex virus-2, equine herpes virus, and influenza, among others (16, 31). The type-I and type-III interferons have been widely used for the treatment of HCV, another pestivirus, and preclinical evaluations have always been performed in humanized mouse models (45, 46). In the present experiments, both these IFN types reduced BVDV infectivity in mice, but did not completely block viral replication. Within the context of our experimental design, we cannot rule out if higher doses would have resulted in a stronger antiviral activity, though we did use a concentration that had already been applied successfully in other similar studies with BALB/c mice (31–34). In a study by Klinkhammer et al. (47), 3 μg of IFN-λ had been used to prevent aerosol infection with influenza virus along with viral transmission. In that work, a single dose of IFN-λ was applied at 18 h pre-infection, in a design similar to the one we used in the first experiment.

IFN-α and IFN-λ had different efficacies when applied either before or after infection, with just a single dose of IFN-λ being more efficient in preventing BVDV infection and with IFN-α providing a better treatment against this virus. IFN-λ receptors are predominantly expressed in epithelial cells; thus, if BVDV uses epithelial cells as a primary replication site, we might conceivably expect an enhanced activity of IFN-λ at that stage. IFN-α receptors are present in a large number of cells (11) and tissues, including blood cells—probably there to prevent viral spread from the initial replication site. For an airborne transmitted virus as VDVB, as the use of IFN-λ may be useful protect the animals at the virus entry site.

Upon analyzing the antiviral capability of IFN-α and -λ on HCV, Marcello et al. (48) proposed that the combined use of both IFNs could achieve better results since the kinetics of the genes inducible by those two IFNs complement each other, which combined activity would generate a better antiviral response. Our study is the first one reporting the antiviral activity of the combined use of IFN-α and -λ *in-vivo*. The use of both IFNs, independently of the scheme, significantly reduced viremia in the infected mice, revealing that increasing the number of applications was more effective than using just one, as expected. Interestingly, IFN-λ applied both before and after infection was an effective treatment, enabling the use of just one cytokine. This result should be considered when designing a trial viral-challenge experiment in cattle.

Our results indicate that infection with 98-124 strain induce a peak of systemic TNF-α at 2 dpi in mice and we observed also that TNF-α was downregulated in most of the IFN-treated group, with significantly lower levels in groups 2 and 5, those being also the ones with the lowest viremia levels. Therefore, we associated the reduced serum concentration of this cytokine with a lower viral replication. We cannot, however, rule out a possible role of these cytokines in modulating the immune response (49), since IFN-α is known to negatively regulate the expression of the proinflammatory cytokines IL-1ß and TNF-α in mice (16), and also suppress IFN-γ production.

In veterinary medicine, because the use of antiviral agents against BVDV has always been focused on treating PI animals, those preparations have not been explored for the possible prevention or treatment of acute infections. We, however, envision the eventual application of IFN-λ in the case of an acute outbreak to reduce viral circulation within the interval between the time of vaccination and onset of protection, potentially closing that window of vulnerability. In the instance where the virus has circulated during the pregnancy period, the pregnant animals can be treated with IFN-λ to prevent transmission, as innate mechanisms of immunity are active even during the fetal period (50). The possibility of expressing bovine IFNs as recombinant proteins (25, 51) furthermore opens up a new scenario for preventing and treating bovine viral infections in the field. Studies in cattle are needed to confirm the activity of IFN-λ in the natural host and to evaluate the time-span of its biological activity, in order to define how long an IFN application can protect against infection in the field.

In conclusion, our results have demonstrated that the BVDV-BALB/c–mouse model constitutes a useful tool to investigate BVDV infection *in-vivo*, and have underscored the efficacy of IFN-λ in reducing BVDV infection *in-vivo*, thus establishing a promising clinical support for the possibility of testing this interferon in bovines.

## Data Availability Statement

All datasets generated for this study are available upon request.

## Ethics Statement

Animal handling, inoculation, and sample collection were performed by trained personnel under the supervision of a veterinarian and following national animal welfare regulations (Protocol N° 6/2017 from CICUAE, INTA).

## Author Contributions

MQ carried out most of the experiments as part of his Ph.D. thesis, with the collaboration of LB, MT, CT, and FM. AC and NC wrote the manuscript. AC conceived the original idea. NC supervised the project.

### Conflict of Interest

The authors declare that the research was conducted in the absence of any commercial or financial relationships that could be construed as a potential conflict of interest.
